# Hepatitis E Virus in Wild Boars and Spillover Infection in Red and Roe Deer, Germany, 2013–2015

**DOI:** 10.3201/eid2301.161169

**Published:** 2017-01

**Authors:** Helena E. Anheyer-Behmenburg, Kathrin Szabo, Ulrich Schotte, Alfred Binder, Günter Klein, Reimar Johne

**Affiliations:** University of Veterinary Medicine Hannover Foundation, Hannover, Germany (H.E. Anheyer-Behmenburg, K. Szabo, G. Klein);; Central Institute of the Bundeswehr Medical Service, Kiel, Germany (H.E. Anheyer-Behmenburg);; German Federal Institute for Risk Assessment, Berlin, Germany (K. Szabo, U. Schotte, A. Binder, R. Johne)

**Keywords:** Hepatitis E virus, wild boar, red deer, roe deer, fallow deer, spillover, zoonoses, viruses, Germany

## Abstract

To determine animal hepatitis E virus (HEV) reservoirs, we analyzed serologic and molecular markers of HEV infection among wild animals in Germany. We detected HEV genotype 3 strains in inner organs and muscle tissues of a high percentage of wild boars and a lower percentage of deer, indicating a risk for foodborne infection of humans.

Hepatitis E is an infection of public health concern, leading to an estimated global disease burden of 3.4 million acute cases, 70,000 deaths, and 3,000 stillbirths per year (*1*). Large disease outbreaks in nonindustrialized countries are mainly caused by drinking water contaminated with hepatitis E virus (HEV) (*2*). In industrialized countries, most cases of hepatitis E are sporadic and suspected to be a result of zoonotic HEV transmission from animals to humans (*3*). The numbers of notified hepatitis E cases have sharply increased in several European countries during recent years (*4*,*5*). Chronic HEV infections among recipients of solid organ transplants pose novel public health concerns (*6*).

HEV belongs to the family *Hepeviridae, *genus *Orthohepevirus*. Its RNA genome comprises 3 open reading frames (ORFs). ORF1 encodes a multifunctional nonstructural polyprotein with methyltransferase and RNA-dependent RNA polymerase genes often used for molecular typing. Human pathogenic HEVs are mainly classified into genotypes 1–4 (*2*,*3*). The camelid HEV genotype 7 was recently detected in a human (*7*), however. Although genotypes 1 and 2 infect only humans, genotypes 3 and 4 are zoonotic and infect different animal species and humans (*2*,*3*,*8*). HEV infection in animals is generally not associated with clinical disease.

The main animal reservoirs for genotype 3 are domestic pigs and wild boars, although infections among other mammals have been described (*2*,*3*,*8*). However, whether these animal species represent true HEV reservoirs or are accidental infections due to spillover events is unclear. In this study, we investigated serologic and molecular evidence of HEV infection in wild boars and different deer species during 2 hunting seasons in a hunting area in Germany.

## The Study

We obtained serum samples from wild boars, roe deer, red deer, and fallow deer during 2 hunting seasons (season A, 2013–2014; season B, 2014–2015) and analyzed them by using an ELISA (ID Screen Hepatitis E Indirect; ID Vet, Grabels, France) for HEV-specific IgG (Technical Appendix Figure 1). Of 339 serum samples, 81 (23.9%) were positive for HEV IgG; results from 1 sample (0.3%) were questionable. Although all wild deer samples tested negative, the proportion of antibody-positive wild boars increased significantly (p = 0.018) from 13 (27.1%; 95% CI 16.55–37.65) of 48 in season A to 68 (51.5%; 95% CI 44.34–58.66) of 132 samples in season B, with a mean antibody prevalence of 45.0% (Table 1). The capability of the ELISA for detection of HEV-specific antibodies in field serum samples from deer was demonstrated by testing of 153 deer serum samples from another hunting area, which led to 3 positive results (data not shown).

**Table 1:** Detection of HEV-RNA and HEV-specific antibodies in wild boars and 3 deer species during 2 hunting seasons in a hunting area in Germany, 2013–2015*

Animal species	HEV-RNA determined by real time RT-PCR, no. positive/no. tested (%)	HEV-specific antibodies determined by ELISA, no. positive/no. tested (%)
Season A, 2013–2014,		
Wild boars	6/95 (6.31)	13/48 (27.08)
Roe deer	2/17 (11.76)	0/8
Red deer	0/25	0/21
Fallow deer	0/2	0/2
Total	8/139 (5.76)	13/79 (16.46)
Season B, 2014–2015		
Wild boars	33/137 (24.09)	68/132 (51.52)
Roe deer	3/61 (4.92)	0/51
Red deer	2/58 (3.45)	0/57
Fallow deer	0/20	0/20
Total	38/276 (13.77)	68/260 (26.15)
Total, 2013–2015	46/415 (11.08)	81/339 (23.89)

*HEV, hepatitis E virus; RT-PCR, reverse transcription PCR.

**Figure 1:**
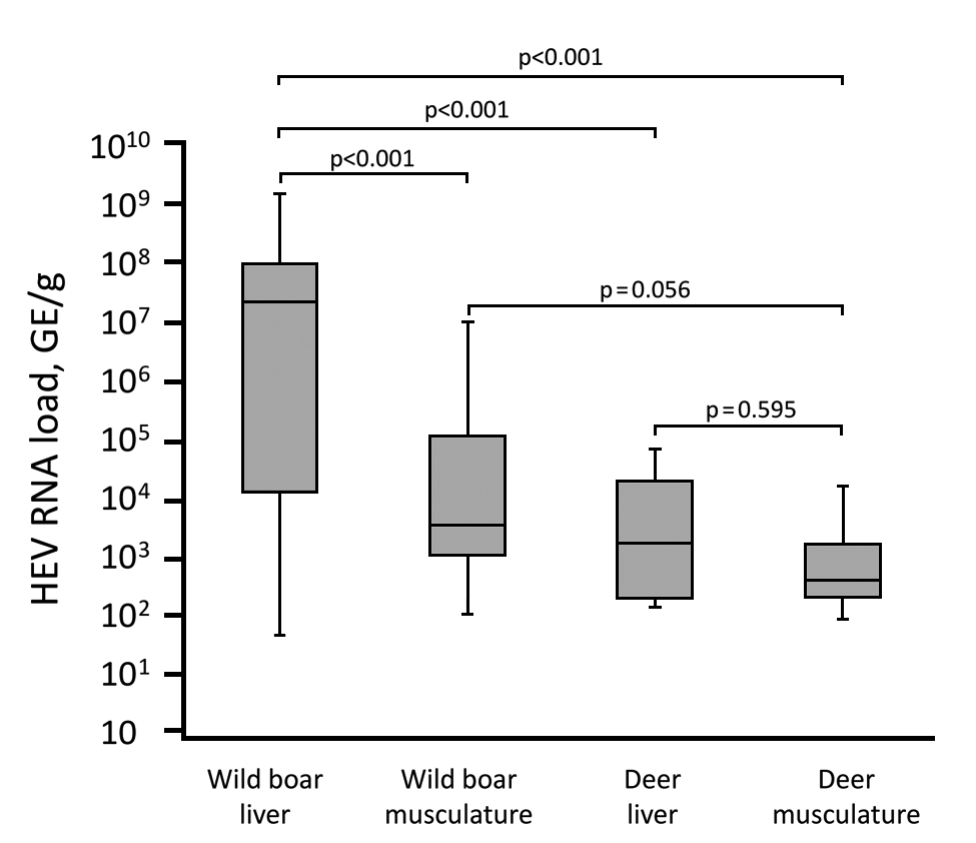
Box plot comparison of HEV RNA load in wild boar and deer specimens from Germany, 2013–2015. Boxes indicate first (bottom) and third quartile; horizontal line within boxes indicate median; error bars indicate minimum and maximum. p values for pairwise comparison of groups are shown. GE, genome equivalents; HEV, hepatitis E virus.

We also tested liver and serum samples from 415 animals for the HEV genome by using real-time reverse-transcription PCR (RT-PCR) (Technical Appendix). HEV RNA was detected in 46 (11.1%) animals: 39 (16.8%) of 232 wild boars (6/95 [6.3%], from season A and 33/137 [24.1%] from season B); 5 (6.4%) of 78 roe deer; and 2 (2.4%) of 83 red deer (Table 1). Testing of all available organs from the HEV-positive wild boars revealed HEV RNA in >89% of the samples. HEV RNA was detected in all tested muscle samples and in most of the other organ samples of HEV-positive deer (Table 2). Comparison of viral loads in the organs revealed significantly higher genome copy numbers in wild boar liver (median 2.26 × 10^7^ genome equivalents[GE]/g]) compared with those for wild boar musculature (median 4.37 × 10^3^ GE/g) or for deer liver (median 2.22 × 10^3^ GE/g) and deer musculature (median 5.25 × 10^2^ GE/g)(Figure 1; Technical Appendix Figure 2). However, the low number of positive deer samples limits the interpretation of the statistical results.

**Table 2:** Organ distribution of HEV RNA in positive-screened wild boars and deer during 2 hunting seasons in a hunting area in Germany, 2013–2015*

Animal species	Sample type, no. positive/no. tested (%)					All
Liver	Musculature	Spleen	Kidney	Serum
Wild boars	26/26 (100.0)	29/35 (82.9)	23/27 (85.2)	16/19 (84.2)	32/34 (94.1)	126/141 (89.4)
Deer	4/5 (80.0)	6/6 (100.0)	2/4 (50.0)	2/4 (50.0)	3/5 (60.0)	17/24 (70.8)
Total	30/31 (96.8)	35/41 (85.4)	25/31 (80.7)	18/23 (78.3)	35/39 (89.7)	143/165 (86.8)

*HEV, hepatitis E virus.

**Figure 2:**
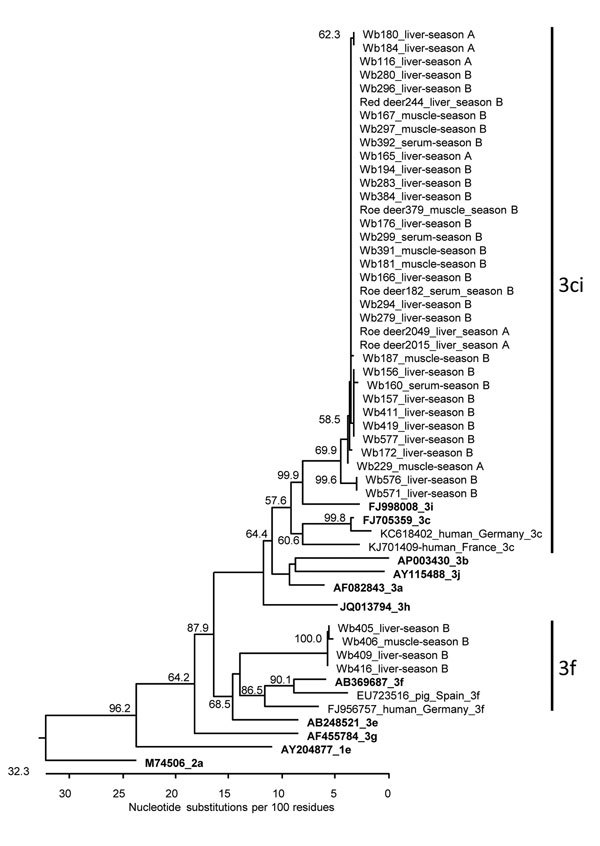
Phylogenetic relationship of HEV sequences derived from wild boars and deer from Germany, 2013–2015. The tree is based on a 280-bp fragment of the ORF1 (RNA-dependent RNA polymerase gene) region. The strain designations, animal species (Wb, wild boar), sample type, and sampling year (season A, 2013–2014; season B: 2014–2015) are indicated for the novel strains. The GenBank accession numbers, the corresponding hosts, the geographic origins and genotypes are indicated for selected additional strains. Reference strains are given in bold; the genotype of the novel strains is indicated at right. Bootstrap values >50% are shown. The tree is scaled in nucleotide substitution units and was constructed using MEGALIGN software (ClustalW [http://www.ebi.ac.uk/Tools/msa/clustalw2/], IUB residue weight table, 1,000 trials and 111 random seeds in bootstrap analysis). HEV, hepatitis E virus; ORF, open reading frame.

A total of 39 of 46 samples were positive in a nested RT-PCR assay targeting the RNA-dependent RNA polymerase gene in the ORF1 (Technical Appendix) that were suitable for sequencing. The amplicons showed nucleotide sequence identities to each other ranging from 73.6% to 100.0%. A phylogenetic tree set up for the samples together with HEV subtype reference strains indicated that most sequences cluster in a clade containing subtypes 3c and 3i (Figure 2). Within this clade, HEV sequences from wild boar and deer from both hunting seasons clustered very closely together. Four sequences from wild boars of season B clustered in genotype 3f. HEV isolates from human hepatitis E cases from Germany clustered near the wild boar and deer HEV sequences (nucleotide sequence identities up to 86.1% to a German 3f strain and 88.2% to a German 3c strain). 

Using a nested RT-PCR assay targeting the methyltransferase gene in the ORF1 (Technical Appendix), we sequenced a PCR product in 18/46 samples. The nucleotide identities of the sequences ranged from 72.7% to 99.6%. A phylogenetic tree again showed grouping into HEV subtype clade 3ci and subtype 3f (Technical Appendix Figure 3). All sequences were deposited in GenBank (accession nos. KX455427–KX455478).

## Conclusions

We detected HEV-RNA and HEV-specific antibodies in a high percentage of wild boars, with a significant difference between the 2 hunting seasons. The detection rates are consistent with previous reports of infection of wild boars in Germany (*9*–*11*). The data underline the high importance of this animal species in the epidemiology of HEV and indicate that wild boars likely represent a persistent reservoir for this virus. The detection of high amounts of HEV RNA in wild boar liver, other organs, and especially in muscle tissue highlights the high risk that HEV can be transmitted to humans through the consumption of meat from these animals that has not been cooked properly.

In contrast, only low percentages of samples from roe deer and red deer tested positive for HEV in our study. Data about HEV infection in wild ruminants in Europe are rare, but some reports have demonstrated HEV infection in several deer species (*12*,*13*). Neumann et al. (*14*) reported serologic and molecular evidence for HEV infection of the indigenous deer species in Germany. We detected HEV RNA in liver, in several organs, and in muscle tissue of the infected deer species. Sequence analysis showed a relationship of HEV from deer with human hepatitis E cases from Germany. In Japan, consumption of deer meat could be linked to acute hepatitis E cases in humans (*15*). Taken together, deer are likely to represent a source of HEV for humans, and consumption of undercooked deer meat should be considered a risk for acquiring HEV infection.

Analysis of the detected HEV sequences indicated that the same strains of genotype clade 3ci circulated in wild boar and deer species. This finding argues against specific HEV strains exclusively circulating in deer species; however, longer sequence parts or whole virus genomes should be analyzed in future studies to support this finding further. The consistently lower HEV RNA and antibody prevalence in deer than in wild boars indicates a primary circulation in wild boars and only accident transmission to deer. The hypothesis of spillover infections of deer is further supported by the consistent lower viral loads in tissues of infected deer. However, other authors classified deer as a true reservoir for HEV (*8*). Further studies investigating more geographic areas over longer time, including the parallel analysis of different animal species, are necessary to unravel the epidemiology and transmission dynamics of HEV in wildlife.

Technical Appendix Materials and methods for analyzing serum samples from wild boars, roe deer, red deer, and fallow deer during 2013–2014 and 2014–2015 for HEV-specific IgG, Germany.

## References

[1] Rein DB, Stevens GA, Theaker J, Wittenborn JS, Wiersma ST. The global burden of hepatitis E virus genotypes 1 and 2 in 2005. Hepatology. 2012;55:988–97. 10.1002/hep.25505 22121109

[2] Johne R, Dremsek P, Reetz J, Heckel G, Hess M, Ulrich RG. Hepeviridae: an expanding family of vertebrate viruses. Infect Genet Evol. 2014;27:212–29. 10.1016/j.meegid.2014.06.024 25050488

[3] Pavio N, Meng XJ, Doceul V. Zoonotic origin of hepatitis E. Curr Opin Virol. 2015;10:34–41. 10.1016/j.coviro.2014.12.006 25588602

[4] Pischke S, Behrendt P, Bock CT, Jilg W, Manns MP, Wedemeyer H. Hepatitis E in Germany—an under-reported infectious disease. Dtsch Arztebl Int. 2014;111:577–83. 25249359 10.3238/arztebl.2014.0577 PMC4174681

[5] Hoofnagle JH, Nelson KE, Purcell RH, Hepatitis E. Hepatitis E. N Engl J Med. 2012;367:1237–44. 10.1056/NEJMra1204512 23013075

[6] Lee GY, Poovorawan K, Intharasongkroh D, Sa-Nguanmoo P, Vongpunsawad S, Chirathaworn C, et al. Hepatitis E virus infection: Epidemiology and treatment implications. World J Virol. 2015;4:343–55. 26568916 10.5501/wjv.v4.i4.343 PMC4641226

[7] Lee GH, Tan BH, Teo EC, Lim SG, Dan YY, Wee A, et al. Chronic infection with camelid hepatitis E virus in a liver transplant recipient who regularly consumes camel meat and milk. Gastroenterology. 2016;150:355–7.e3. 10.1053/j.gastro.2015.10.048 26551551

[8] Van der Poel WH. Food and environmental routes of Hepatitis E virus transmission. Curr Opin Virol. 2014;4:91–6. 10.1016/j.coviro.2014.01.006 24513966

[9] Oliveira-Filho EF, Bank-Wolf BR, Thiel HJ, König M. Phylogenetic analysis of hepatitis E virus in domestic swine and wild boar in Germany. Vet Microbiol. 2014;174:233–8. 10.1016/j.vetmic.2014.09.011 25287630

[10] Schielke A, Ibrahim V, Czogiel I, Faber M, Schrader C, Dremsek P, et al. Hepatitis E virus antibody prevalence in hunters from a district in Central Germany, 2013: a cross-sectional study providing evidence for the benefit of protective gloves during disembowelling of wild boars. BMC Infect Dis. 2015;15:440. 10.1186/s12879-015-1199-y 26493830 PMC4619084

[11] Schielke A, Sachs K, Lierz M, Appel B, Jansen A, Johne R. Detection of hepatitis E virus in wild boars of rural and urban regions in Germany and whole genome characterization of an endemic strain. Virol J. 2009;6:58. 10.1186/1743-422X-6-58 19442307 PMC2689194

[12] Di Bartolo I, Ponterio E, Angeloni G, Morandi F, Ostanello F, Nicoloso S, et al. Presence of Hepatitis E virus in a red deer (*Cervus elaphus*) population in Central Italy. [Epub 2015 Apr 19]. Transbound Emerg Dis. 2015. 10.1111/tbed.12353 25892400

[13] Kubankova M, Kralik P, Lamka J, Zakovcik V, Dolanský M, Vasickova P. Prevalence of hepatitis E virus in populations of wild animals in comparison with animals bred in game enclosures. Food Environ Virol. 2015;7:159–63. 10.1007/s12560-015-9189-1 25771162

[14] Neumann S, Hackl SS, Piepenschneider M, Vina-Rodriguez A, Dremsek P, Ulrich RG, et al. Serologic and molecular survey of hepatitis E virus in German deer populations. J Wildl Dis. 2016;52:106–13. 10.7589/2014-12-282 26528571

[15] Tei S, Kitajima N, Takahashi K, Mishiro S. Zoonotic transmission of hepatitis E virus from deer to human beings. Lancet. 2003;362:371–3. 10.1016/S0140-6736(03)14025-1 12907011

