# Supplementary material for: Hepatitis E Virus in Wild Boars and Spillover Infection in Red and Roe Deer, Germany, 2013–2015
Source: Emerg Infect Dis. 2017 Jan;23(1):130–3. doi: 10.3201/eid2301.161169 (PMC5176221; doi:10.3201/eid2301.161169)
Supplement: Technical Appendix — Materials and methods for analyzing serum samples from wild boars, roe deer, red deer, and fallow deer during 2013–2014 and 2014–2015 for HEV-specific IgG, Germany. [file 16-1169-Techapp-s1.pdf]

Article DOI: http://dx.doi.org/10.3201/eid2301.161169

Hepatitis E Virus in Wild Boars and Spillover Infection in Red and Roe Deer, Germany, 2013–2015

Technical Appendix

Materials

During the 2 hunting seasons (season A: November 2013–January 2014; season B: November 2014–January 2015), samples of 415 wild animals (wild boars [*Sus scrofa*], roe deer [*Capreolus capreolus*], red deer [*Cervus elaphus*], fallow deer [*Dama dama*]) were collected from liver, spleen, kidney, muscle, and serum (not all sample types were available in all animals). All animals belonged to free-ranging populations living in a military training area of the German armed forces (53°36′34 N, 14°02′36 E) in Mecklenburg–Western Pomerania, Germany (Figure 1). The sampling took place subsequently after the battue (game were driven toward hunters by beaters).

Methods

ELISA

Serum samples were analyzed for hepatitis E virus (HEV)–specific antibodies by an indirect ELISA based on a recombinant HEV genotype 3 capsid protein and an anti-multispecies IgG–horseradish peroxidase conjugate (ID Screen Hepatitis E Indirect; ID Vet, Grabels, France). In addition to the control samples included in the kit, HEV-positive serum samples from deer were used as control samples. Test procedures were performed, and results were interpreted according to the manufacturer’s instructions. The optical density was measured at 450-nm wavelength by a plate reader (Sunrise, Tecan Group Ltd, Männedorf, Switzerland).

RNA Extraction

Liver samples from all animals were screened by an HEV-specific real-time reverse transcription PCR (RT-PCR); if those were not available, serum samples were used. 120 mg

portions of the samples were suspended in 700 μL of phosphate-buffered saline (PBS) and homogenized by using a 5-mm-diameter steel ball in a Tissue Lyser (QIAGEN, Hilden, Germany) for 210 seconds at 30 Hz. After centrifugation at 2,500 × *g* for 15 min, 50 μL of supernatant was mixed with 350 μL of Buffer RLT (QIAGEN) before centrifugation through a QIA-Shredder (QIAGEN) at 16,000 × *g* for 180 s. RNA was extracted by using the QIAsymphony RNA kit by using a 2-fold concentrated DNase in a QIAsymphony device according to the manufacturer's protocol. The elution volume was 50 μL. Nucleic acids were extracted from 350 µl serum using the QIAsymphony DSP Virus/Pathogen Kit in a QIAsymphony device according to the manufacturer's instructions. The elution volume was 60 μL.

Real-Time RT-PCR

An HEV-specific RT-qPCR was performed with the QuantiTect Probe RT-PCR Kit (QIAGEN) by using 5 μL of sample RNA in a total volume of 20 μL. Primers and probe were used according to the method of Jothikumar et al. (*1*). Reverse transcription was performed for 30 min at 50°C, followed by denaturation for 15 min at 95°C and 45 cycles each with 30 s at 94°C, 30 s at 55°C and 30 s at 72°C. Quantification of all available organs from positive screened animals was performed by using a dilution series of 10^3 to 10^{14} copies of in vitro-transcribed RNA.

RT-PCR and Sequencing

Two nested RT-PCR assays targeting the RNA-dependent RNA polymerase region (RdRp) (*2*) and methyltransferase region (*3*) within open reading frame 1 were performed for subsequent sequence analysis. The One-Step RT-PCR Kit (QIAGEN) was used for the RT-PCR and the TaKaRa Ex Taq (Takara Bio Europe S.A.S., Saint-Germain-en-Laye, France) for the nested PCR. The nested PCR products with sizes of 331 bp (*2*) or 286 bp (*3*) were separated on ethidium bromide–stained 1.5% agarose gels and visualized by ultraviolet light. The amplicons were purified by using the QIAquick DNA purification kit (QIAGEN) and Sanger sequencing was carried out by an external company (Eurofins Genomics, Ebersberg, Germany) by using the nested PCR primers. Sequences (excluding primer sequences) were deposited in GenBank with accession nos. KX455427–KX455478.

Sequence Analysis

Nucleotide sequences were analyzed using the DNASTAR software package (Lasergene, Madison, WI, USA), and compared with HEV sequences available in the NCBI GenBank database (http://www.ncbi.nlm.nih.gov) by using the BLASTn facility as well as with HEV subtype reference strains (*4*). Phylogenetic trees were constructed on the basis of the nucleotide sequences by using a neighbor-joining method implemented in the MegAlign module of the DNASTAR software package (Lasergene) with the parameters: ClustalW method, IUB residue weight table and a bootstrap analysis with 1,000 trials and 111 random seeds.

Statistical Analysis

The median, minimum, maximum, and first and third quartiles were calculated and used for generation of boxplot figures using Excel (Microsoft Office Professional 2010, Microsoft Corp., Redmond, WA, USA). The differences of estimated prevalences per season were tested for significance with the 2-sample *z* test (http://epitools.ausvet.com.au/content.php?page = z-test-2), differences of HEV-RNA load in organ samples were tested with the logarithm of GE/g using 2-sample *t*test for summary data (http://epitools.ausvet.com.au/content.php?page = 2-sample-t-test). All analyses were done by using EpiTools epidemiologic calculators (Ausvet Pty Ltd., available at http://epitools.ausvet.com.au).

References

1. Jothikumar N, Cromeans TL, Robertson BH, Meng XJ, Hill VR. A broadly reactive one-step real-time RT-PCR assay for rapid and sensitive detection of hepatitis E virus. J Virol Methods. 2006;131:65–71. PubMed http://dx.doi.org/10.1016/j.jviromet.2005.07.004

2. Johne R, Plenge-Bönig A, Hess M, Ulrich RG, Reetz J, Schielke A. Detection of a novel hepatitis E-like virus in faeces of wild rats using a nested broad-spectrum RT-PCR. J Gen Virol. 2010;91:750–8. PubMed http://dx.doi.org/10.1099/vir.0.016584-0

3. Vollmer T, Diekmann J, Johne R, Eberhardt M, Knabbe C, Dreier J. Novel approach for detection of hepatitis E virus infection in German blood donors. J Clin Microbiol. 2012;50:2708–13. PubMed http://dx.doi.org/10.1128/JCM.01119-12

4. Smith DB, Simmonds P, Izopet J, Oliveira-Filho EF, Ulrich RG, Johne R, et al. Proposed reference sequences for hepatitis E virus subtypes. J Gen Virol. 2016;97:537–42. PubMed http://dx.doi.org/10.1099/jgv.0.000393

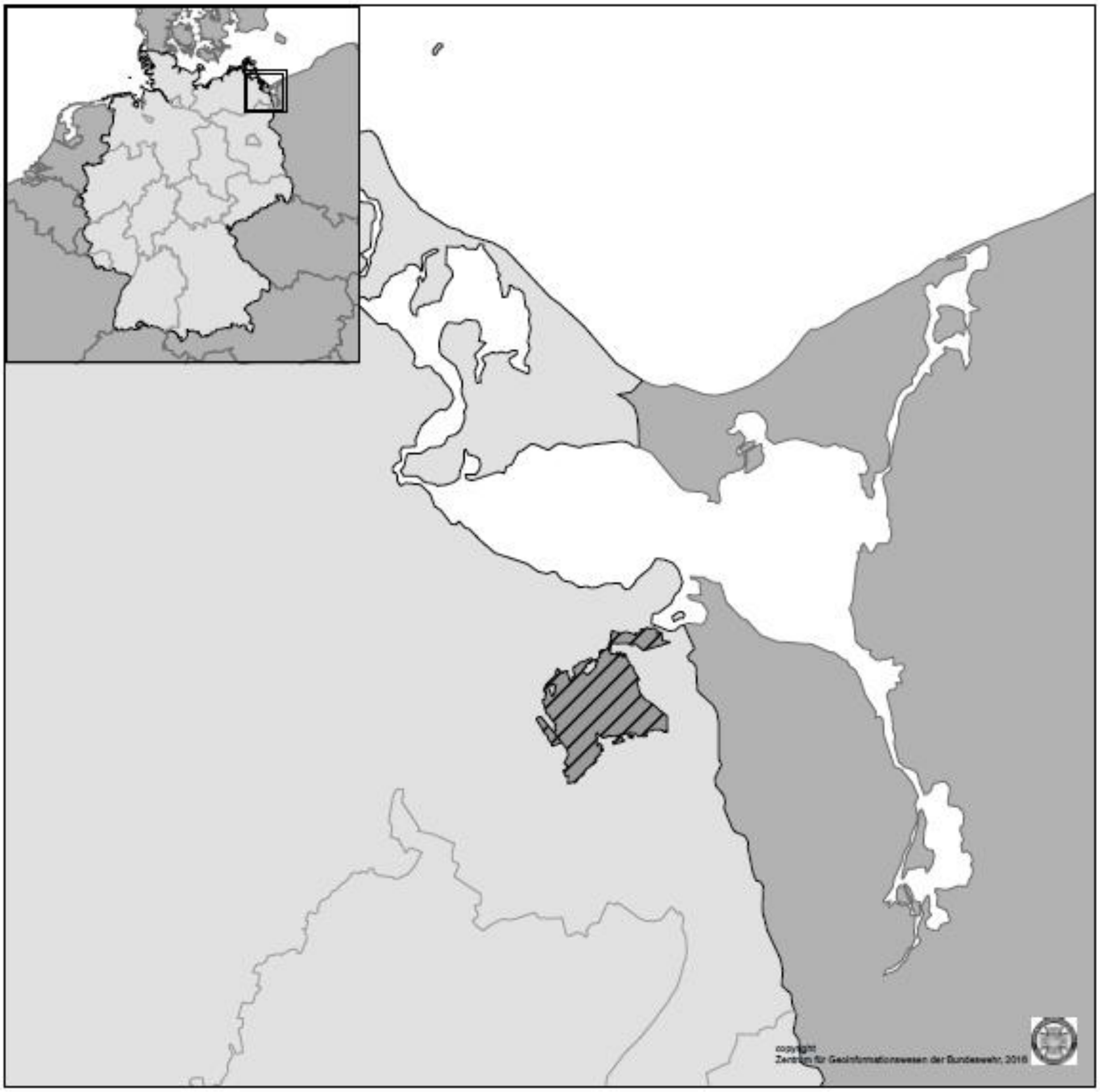

Technical Appendix Figure 1. Location of the hunting area within Germany where wild boars, roe deer, red deer, and fallow deer were sampled for hepatitis E virus, Germany, 2013–2015 (indicated by cross-hatching).

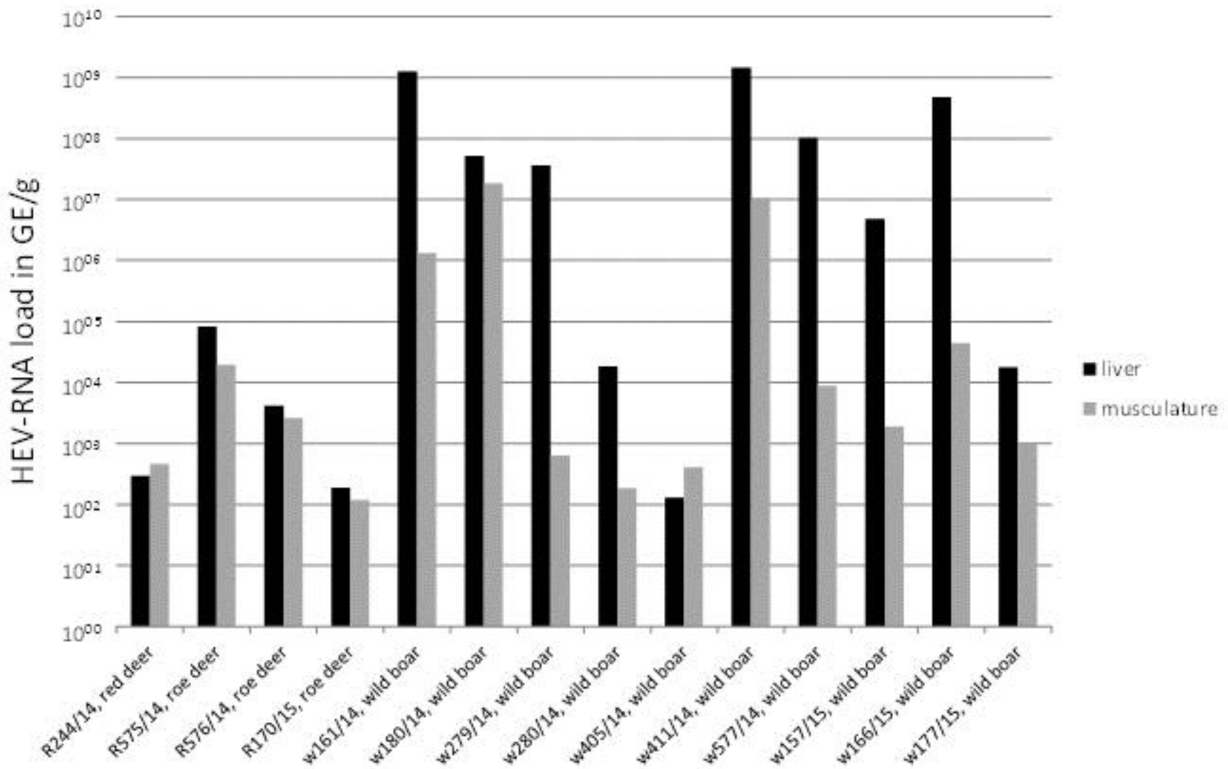

Technical Appendix Figure 2. Hepatitis E virus RNA load in liver and muscle specimens of individual deer and wild boars, Germany, 2013–2015.

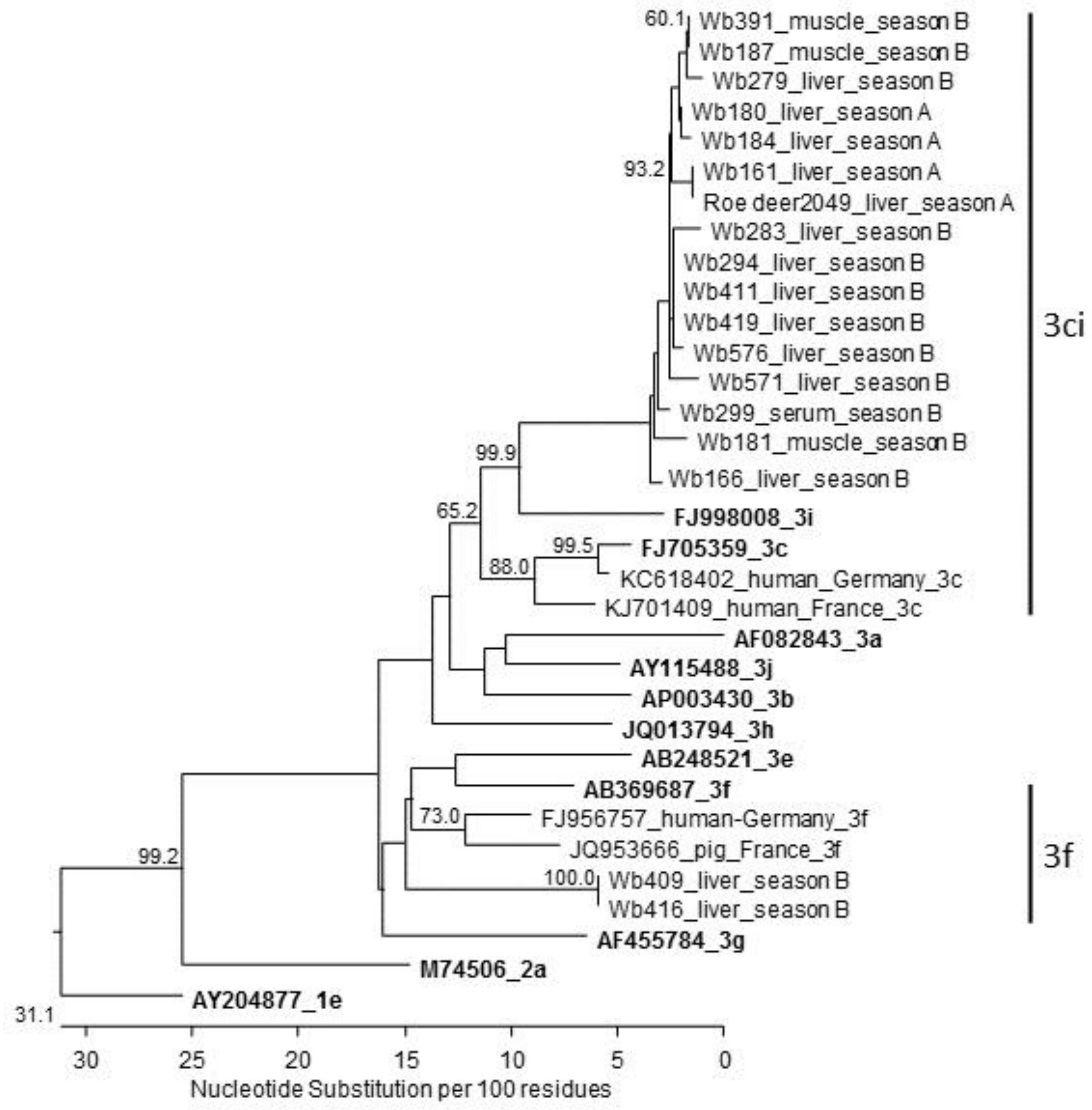

Technical Appendix Figure 3. Phylogenetic relationship of hepatitis E virus sequences derived from wild boars (wb) and deer, Germany, 2013–2015. The tree is based on a 242-bp fragment of the open reading frame 1 (methyltransferase gene) region. The strain designations, animal species, sample type, and sampling year (season A: 2013–2014; season B: 2014–2015) are indicated for the novel strains. The GenBank accession numbers, the corresponding hosts, the geographic origins and genotypes are indicated for selected additional strains. Reference strains are given in bold, and the genotype of the novel strains is indicated on the right. Bootstrap values >50% are shown. The tree is scaled in nucleotide substitution units and was constructed by using the MEGALIGN module of the DNASTAR software package (Lasergene; parameters: ClustalW, IUB residue weight table, 1,000 trials and 111 random seeds in bootstrap analysis).
